# Mouse Y-Encoded Transcription Factor *Zfy2* Is Essential for Sperm Formation and Function in Assisted Fertilization

**DOI:** 10.1371/journal.pgen.1005476

**Published:** 2015-12-31

**Authors:** Yasuhiro Yamauchi, Jonathan M. Riel, Victor Ruthig, Monika A. Ward

**Affiliations:** Institute for Biogenesis Research, John A. Burns School of Medicine, University of Hawaii, Honolulu, Hawaii, United States of America; University of Pennsylvania, UNITED STATES

## Abstract

Spermatogenesis is a key developmental process allowing for a formation of a mature male gamete. During its final phase, spermiogenesis, haploid round spermatids undergo cellular differentiation into spermatozoa, which involves extensive restructuring of cell morphology, DNA, and epigenome. Using mouse models with abrogated Y chromosome gene complements and Y-derived transgene we identified Y chromosome encoded *Zfy2* as the gene responsible for sperm formation and function. In the presence of a *Zfy2* transgene, mice lacking the Y chromosome and transgenic for two other Y-derived genes, *Sry* driving sex determination and *Eif2s3y* initiating spermatogenesis, are capable of producing sperm which when injected into the oocytes yield live offspring. Therefore, only three Y chromosome genes, *Sry*, *Eif2s3y* and *Zfy2*, constitute the minimum Y chromosome complement compatible with successful intracytoplasmic sperm injection in the mouse.

## Author Summary

The mammalian Y chromosome was once thought to be a genetic wasteland with testis determinant *Sry* being the only gene of importance. We now know that there are many genes on this chromosome crucial for male reproduction but their specific roles are often undefined. Here, we investigated the function of the Y chromosome gene *Zfy2* during a final step of male gamete formation. We demonstrated that *Zfy2* is responsible for allowing sperm precursor cells, haploid round spermatids, to undergo transformation into spermatozoa, and that these sperm are capable of yielding live offspring when injected into the oocytes. Thus, we identified a novel role of the *Zfy2* gene during spermatogenesis and fertilization. Considering that in human sperm formation is a prerequisite for male infertility treatment using assisted reproduction technologies, our finding bear translational significance.

## Introduction

Y chromosome has always been considered a symbol of maleness as it encodes testis determining gene *Sry* which acts in the developing gonads and induces the development of testes rather than ovaries [1–3]. Mammalian Y chromosomes encode a number of other genes most of which are thought to be involved in various aspects of male reproduction, and other playing roles of broadly expressed regulators of transcription, translation and protein stability [4]. In spite of these clearly important functions, the knowledge linking the roles of specific Y chromosome genes to specific reproductive processes remains limited.

**Fig 1:**
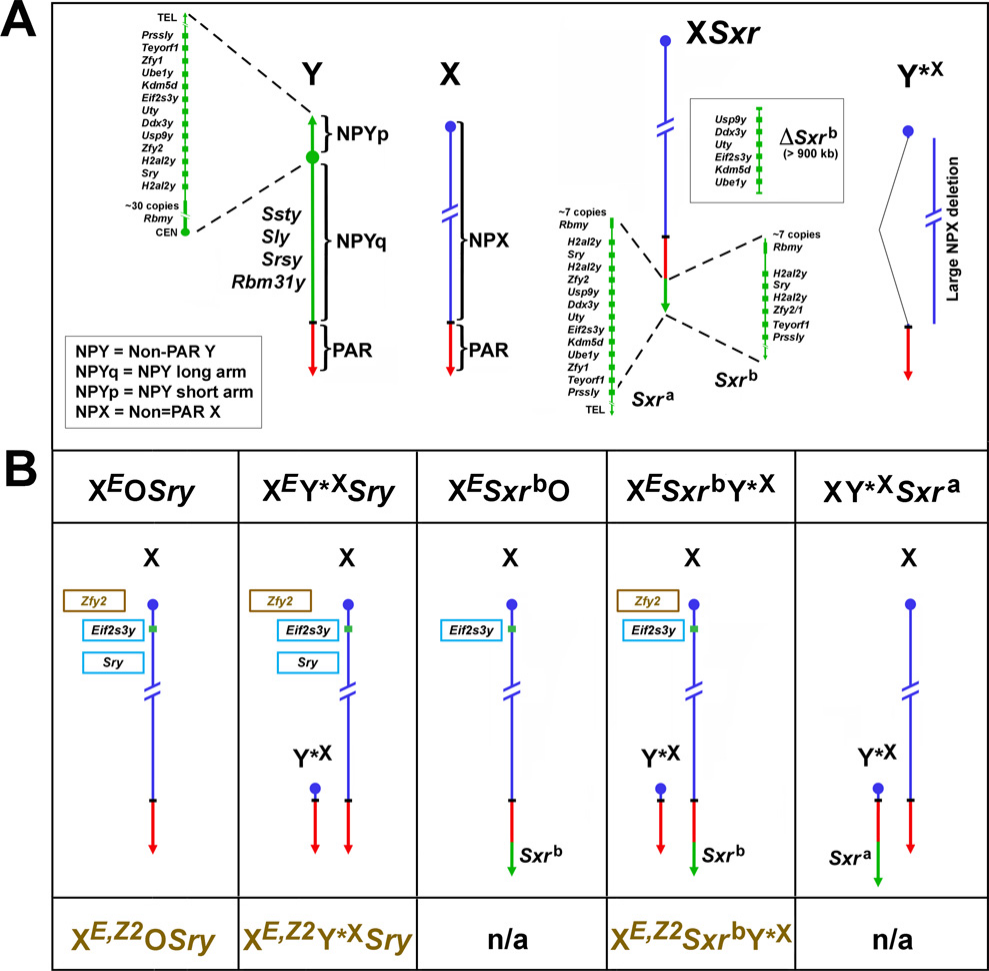
Mouse X and Y chromosomes, variant sex chromosomes, and mouse genotypes relevant to this study. (A) The mouse Y chromosome contains ~90 Mb of male specific DNA and ~0.7 Mb constituting the pseudoautosomal region (PAR) situated at the end of the long arm. The PAR is the region of homology with the X that mediates pairing and recombination between the X and Y in normal males. The remaining non-pairing male specific part of Y (NPY) contains several genes and gene families. On the short arm (NPYp), there are single**-**copy genes: *Prssly*, *Teyorf1*, *Uba1y*, *Smcy/Kdm5d*, *Eif2s3y*, *Uty*, *Dby/Ddx3y*, *Usp9y*, *Sry*, duplicated gene *Zfy* (*Zfy1* and *2*), duplicated gene *H2al2y*, and a multi-copy gene *Rbmy*. The non-pairing region of the long arm (NPYq), representing ~90% of all NPY, contains mostly repetitive sequences, and encodes multiple copies of 5 distinct genes that are expressed in spermatids: *Ssty1* and *Ssty2*, *Sly*, *Srsy*, *Rbm31y* [6]. Y*^X^ is an X chromosome derivative encoding PAR, X centromere and near centromeric region. *Sxr* ^a^ is a sex reversal variant Tp(Y)1Ct^*Sxr-a*^ encoding almost intact NPYp complement but with *Rbmy* gene family reduced. *Sxr* ^b^ *is a Sxr* ^a^ derivative with a 1.3 Mb deletion that has removed the majority of the NPYp gene complement and created a *Zfy2/1* fusion gene. (B) The mice used in this study and their Y chromosome contribution. The X chromosome located *Eif2s3y* and autosomally located *Sry* transgenes, are shown in light blue frames. The *Zfy2* transgene, shown in brown frame, is located on the X chromosome in the *Hrpt* locus in close proximity to the *Eif2s3y* transgene. The genotype designations without the *Zfy2* transgene are shown above the diagrammatic representation of sex chromosomes and with the *Zfy2* transgene below them (brown font). *Sxr* ^a^ and *Sxr* ^b^ gene content is shown in A. n/a = mice with transgenic *Zfy2* addition were either not produced or not examined in this study.

We recently investigated spermatogenesis progression and germ cell function in male mice with significantly abrogated Y chromosome complements [5]. We have shown that males with the Y chromosome contribution provided by two transgenes, the testis determinant *Sry* and the spermatogonial proliferation factor *Eif2s3y* (Fig 1B, X^*E*^O*Sry* and X^*E*^Y*^X^ *Sry*) have meiotic and postmeiotic arrest, the rare spermatids present in the testes do not elongate, and sperm are not formed. When round spermatids from these males were injected into the oocytes, live mouse progeny were obtained. The success of round spermatid injection (ROSI) was low, with less than 10% of transplanted embryos developing to live offspring. Interestingly, when the *Sry* transgene was replaced with the Y chromosome derived sex reversal factor *Sxr* ^b^, encoding for *Sry*, *H2al2y*, *Prssly*, *Teyorf1*, *Rbmy* gene cluster, and *Zfy2/1* fusion gene (Fig 1A, *Sxr* ^b^) the resulting males (Fig 1B, X^*E*^ *Sxr* ^*b*^O and X^*E*^ *Sxr* ^*b*^Y*^X^) had more advanced spermatid development with clear elongation of these cells, occasional appearance of sperm, and increased ROSI efficiency.

These findings indicated that a gene/s encoded within *Sxr* ^b^ plays a role in spermiogenesis progression and germ cell function. Here, we identify *Zfy2* as the gene responsible. We present the evidence that the Y chromosome gene *Zfy2* promotes sperm morphogenesis, improves ROSI success, and is necessary for a formation of sperm capable of yielding live offspring after intracytoplasmic injection into the oocytes.

## Results

### Sperm from X^*E*^ *Sxr* ^*b*^O and X^*E*^ *Sxr* ^*b*^Y*^X^ males are not functional in assisted fertilization

The presence of *Sxr* ^b^ enables spermatid elongation in X^*E*^ *Sxr* ^*b*^O and X^*E*^ *Sxr* ^*b*^Y*^X^ males, with occasional development of mature testicular sperm [5,7] (S1 Fig). To test for the ability of these testicular sperm to participate in successful assisted fertilization and embryo development, we performed intracytoplasmic sperm injection (ICSI). No live offspring were obtained from X^*E*^ *Sxr* ^*b*^O males (n = 4 males, 0/94 fetuses from embryos transferred), while ICSI with sperm from X^*E*^ *Sxr* ^*b*^Y*^X^ males yielded a single fetus (n = 5 males, 1/84 fetuses from embryos transferred). Thus, sperm from X^*E*^ *Sxr* ^*b*^O and X^*E*^ *Sxr* ^*b*^Y*^X^ males are virtually not successful in assisted fertilization.

A possible reason for the lack of live offspring from these sperm could be sperm diploidy. We have previously shown that the great majority (86%) of round spermatids from X^*E*^ *Sxr* ^*b*^O males were diploid while the opposite was true for X^*E*^ *Sxr* ^*b*^Y*^X^ males, in which haploid round spermatids predominated (71%) [5]. To test whether testicular sperm from these males carried doubled DNA content we performed zygotic chromosome analysis after sperm injection (Table 1, S2 Fig). This analysis demonstrated that only about one-fourth of the embryos obtained after ICSI with sperm from X^*E*^ *Sxr* ^*b*^O males were diploid (26%, 12/46, Table 1); the remaining zygotes were triploid and thus presumably derived from diploid sperm. Zygotes obtained after ICSI with sperm from X^*E*^ *Sxr* ^*b*^Y*^X^ males were predominantly diploid (64%, 9/14, Table 1). These data support that while sperm diploidy might be responsible for the lack of ICSI success with X^*E*^ *Sxr* ^b^O, it is not likely the case with X^*E*^ *Sxr* ^*b*^Y*^X^ males. We have shown earlier that testicular sperm from males carrying the Y chromosome derived sex reversal factor *Sxr* ^a^ (Fig 1A & 1B, *Sxr* ^a^, XY*^X^ *Sxr* ^*a*^) are haploid [8]. Thus, functional deficiency of sperm from X^*E*^ *Sxr* ^*b*^Y*^X^ males is likely due to lack of one or more Y chromosome genes that are present in XY*^X^ *Sxr* ^*a*^ and not in X^*E*^ *Sxr* ^*b*^Y*^X^.

**Table 1:** Zygotic chromosome analysis after ICSI with sperm from X^*E*^ *Sxr* ^b^O and X^*E*^ *Sxr* ^*b*^Y*^X^ males.

Male genotype	ICSI zygotes	
	Diploid % (No)	Triploid % (No)
X^*E*^O*Sry*	no sperm	
X^*E*^Y*^X^ *Sry*	no sperm	
X^*E*^ *Sxr* ^b^O	26 (12/46)^a^ ^,^ ^c^	74 (34/46)
X^*E*^ *Sxr* ^b^Y*^X^	64 (9/14)^b^	36 (5/14)
XY	100 (21/21)	0 (0/21)

Statistical significance (Fisher's Exact Test):

^a^ P<0.001

^b^ P<0.01 vs. XY

^c^ P<0.05 vs. X^*E*^ *Sxr* ^b^Y*^X^. For explanation of male genotypes see Fig 1, S1 Table, and Text.

Overall, the data demonstrate that testicular sperm from X^*E*^ *Sxr* ^*b*^O and X^*E*^ *Sxr* ^*b*^Y*^X^ males are not functional in assisted fertilization and that this may reflect lack of certain Y gene/s.

### Addition of *Zfy2* to X^*E*^Y*^X^ *Sry* males enables spermatid elongation

We next investigated which of the *Sxr* ^b^ genes is responsible for spermatid elongation. The gene content of *Sxr* ^*b*^ is represented by few copies of *Rbmy*, two copies of *H2al2y*, one copy of *Sry*, *Prssly* and *Teyorf1*, and a *Zfy2/1* fusion gene spanning the *Sxr* ^*b*^ deletion breakpoint (Fig 1A). *Rbmy* appears early during spermatogenesis and is not expressed, and certainly not translated, after the zygotene stage [9]. *H2al2y* has been shown to be expressed late during spermiogenesis [10,11] and *Sry* transcripts in adult gonads are thought to be aberrant and not translatable [12,13]. *Prssly* and *Teyorf1* are newly discovered genes [6] whose expression has not been characterized; we were not aware of these genes existence when the study was initiated. Based on the known expression pattern, we excluded *Rbmy*, *H2al2y*, and *Sry* as the candidates for ensuring sperm head and tail morphogenesis in males with *Sxr* ^b^, and focused our attention on the *Zfy2/1* fusion gene.

Postnatal expression of *Zfy1* and *Zfy2* is restricted to spermatogenic cells [14–16]. Both genes are first expressed in the testis around the time when cells enter meiosis. They then undergo transcriptional silencing as the cells enter pachynema. The expression is reactivated in secondary spermatocytes and continues postmeiotically [11,17]. In round spermatids there is a clear predominance of *Zfy2* transcripts in Y-bearing round spermatids; this strong expression appears because of the activity of 'acquired' strong *Cypt*-derived spermatid-specific promoter driving *Zfy2* expression [17]. The CYPT exon of *Zfy2*, encoding the *Cypt1* promoter is thought to be derived from the *Cypt1* gene [14,17], which belongs to the CYPT spermatid-specific gene family [18]. The expression of *Zfy1*, which lacks the *Cypt* promoter, is limited at the round spermatid stage. The *Zfy2/1* fusion gene is driven by the *Cypt* promoter and is strongly expressed in spermatids. Our expectation therefore was that *Zfy2*, and not *Zfy1*, would mimic the effect of *Sxr* ^b^.

**Fig 2:**
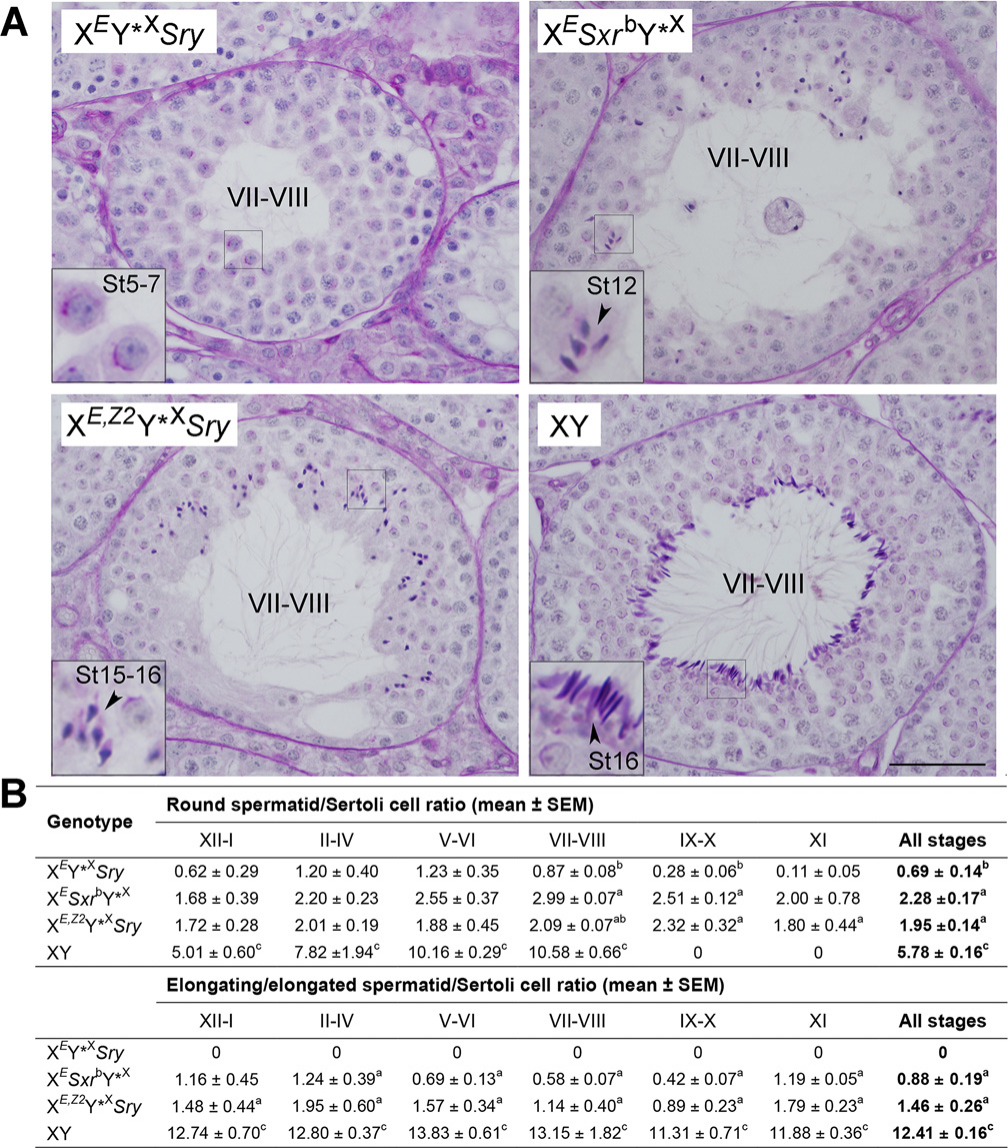
Histology analysis. (A) Exemplary tubules of stage VII-VIII testis sections. X^*E*^Y*^X^ *Sry* males have meiotic and post-meiotic arrest that only occasionally allow formation of round spermatids that do not develop beyond step 7 of spermatid development. In X^*E*^ *Sxr* ^b^Y*^X^ spermatid elongation is observed but usually ceases at step 11–12, with few occurrences of more advanced stages. In X^*E*,*Z2*^Y*^X^ *Sry* males spermatogenesis is progressing with good spermatid elongation and many spermatids developing to step 15–16; these elongated spermatids are morphologically abnormal, which is expected from males lacking NPYq genes [19]. Tubule stages are shown in Roman numerals and steps of spermatid development (St) in Arabic numerals. Bar = 50 μm; insets = x3 magnification. See also S3 Fig emphasizing spermatid at step 7–8. (B) Quantitative analysis of spermatogenesis progression. For each male 10 tubules were examined per stage and the numbers of round spermatid (steps 1–8), elongating/ed spermatid (steps 9–16), and Sertoli cells were counted. The data are expressed as spermatid/Sertoli cell ratios. In wild-type males no round spermatids are present in stages IX-XI so those observed in males with limited Y gene complement represent 'delayed spermatids'. Statistical significance (t-test): ^a^ different than X^*E*^Y*^X^ *Sry*; ^b^ different than X^*E*^ *Sxr* ^b^Y*^X^; ^c^ different than all other. Three males per genotypes were included in the analysis. For explanation of male genotypes see Fig 1, S1 Table, and text.

To assess *Zfy2* role in spermatogenesis progression we investigated testis histology in X^*E*^Y*^X^ *Sry* males transgenic for *Zfy2* (Fig 2 and S3 Fig). These males are subsequently called X^*E*,*Z2*^Y*^X^ *Sry* (Fig 1B, S1 Table). While in X^*E*^Y*^X^ *Sry* males spermatid development did not progress beyond the round spermatid stage, in X^*E*,*Z2*^Y*^X^ *Sry* males spermatids elongated (Fig 2A). The elongated, condensed spermatids were more frequently observed in X^*E*,*Z2*^Y*^X^ *Sry* than in X^*E*^ *Sxr* ^b^Y*^X^ males; in the latter genotype elongation often ceased earlier (at step 12–13) and the spermatid nuclei were less compacted, with lighter staining pattern. Quantitative analysis of spermatogenesis progression (Fig 2B) demonstrated that X^*E*,*Z2*^Y*^X^ *Sry* had more round spermatids than X^*E*^Y*^X^ *Sry* (~2.8-fold increase), reaching a level similar to that observed with X^*E*^ *Sxr* ^b^Y*^X^ males, but less than in wild-type controls. The number of elongating/elongated spermatids in X^*E*,*Z2*^Y*^X^ *Sry* and X^*E*^ *Sxr* ^b^Y*^X^ was not significantly different, and ~10-fold lower than in wild-type controls. In the quantitative analysis of testis sections we did not distinguish between the elongating and elongated spermatids because the abnormal morphology of developing spermatids, which ultimately resulted in severely morphologically abnormal headshape of testicular and epididymal sperm, made such distinction impossible.

### Sperm from X^*E*^Y*^X^ *Sry* males have headshape defects

**Fig 3:**
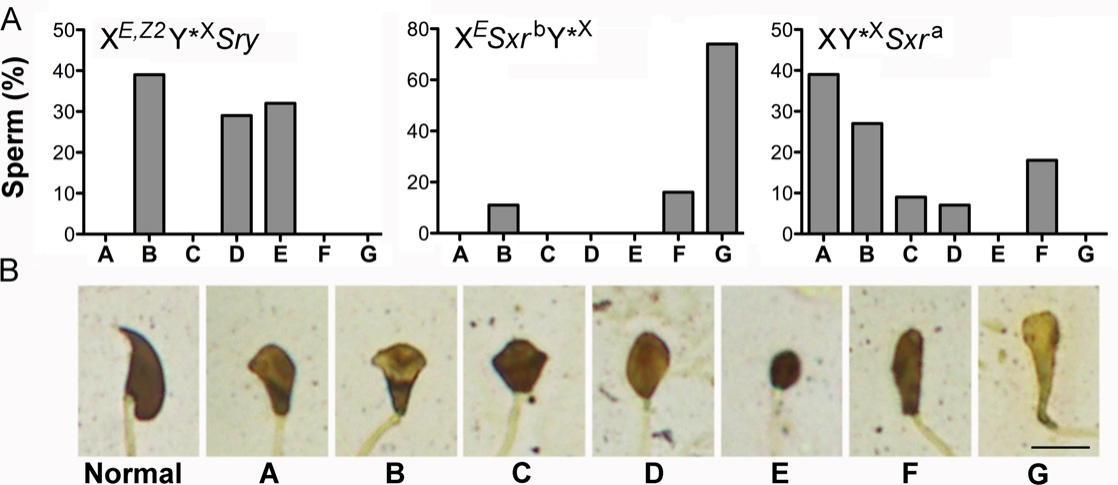
Sperm headshape analysis. (A) The distribution of specific headshape defect categories among testicular sperm from X^*E*,*Z2*^Y*^X^ *Sry* (n = 41 sperm from 3 males), X^*E*^ *Sxr* ^b^Y*^X^ (n = 19 sperm from 2 males), and XY*^X^ *Sxr* ^a^ (44 sperm from 3 males) males. (B) The categories of headshape defects. Normal: represents a normal shape of testicular sperm head. A ("dolphin"): sperm head is elongated and has some curvature reminiscent of crescent shape typical for mouse sperm, small hooked tip can be differentiated. B ("mushroom"): sperm head is elongated but the curvature is not present, hint of a hooked tip can sometimes be observed. C ("cupcake"): sperm head is no longer elongated, the caudal side is wider than in A and B categories, and opens up to a wide dorsal side; hint of a hooked tip can be sometimes be seen. D ("egg"): sperm head has an oval shape with no mark of a hooked tip. The head is less elongated than in category B; E ("ball"): sperm head has a round shape with no hint of a hooked tip, is smaller than in all other categories, and is strongly stained indicative of high DNA condensation; F ("drumstick"): sperm head is elongated, has no traces of a hooked tip, is longer and thinner than in category A&B but shorter than in G. G ("club"): sperm head is clearly elongated with no hint of a hooked tip, and very poorly condensed. Scale = 5 μm.

Sperm from X^*E*,*Z2*^Y*^X^ *Sry* males were also observed in live epididymal and testicular cell suspension, with and on silver stained testicular cell spreads (S4 Fig). The epididymal sperm were extremely rare; only few immotile sperm were noted in 4 out of 5 males. The headshape of both testicular and epididymal sperm was abnormal, as expected from males lacking Y chromosome long arm [19]. To characterize structural sperm defects in more detail we performed the analysis of sperm headshape on silver stained testicular cell spreads (Fig 3). Only sperm with fully developed tails were included in this analysis. In XY males, the great majority of testicular sperm were normal (84%, 31/37), with remaining having slight headshape defects, comparable to those noted earlier in epididymal sperm [19]. In XY*^X^ *Sxr* ^a^, X^*E*^ *Sxr* ^b^Y*^X^ and X^*E*,*Z2*^Y*^X^ *Sry* males all sperm were morphologically abnormal. We divided the observable headshape defects into 7 categories (A-G) (Fig 3B) and quantified their incidence (Fig 3A). In X^*E*,*Z2*^Y*^X^ *Sry* males sperm heads were either oval or rounded in shape, with no hint of a hooked tip, and frequently highly condensed (categories D and E, 61%), or were elongated with no curvature reminiscent of crescent shape typical for mouse sperm, and occasional hint of a hooked tip (category B, 39%). Sperm in XY*^X^ *Sxr* ^a^ males, which similarly as X^*E*,*Z2*^Y*^X^ *Sry* lack Y chromosome long arm, had better developed heads than in X^*E*,*Z2*^Y*^X^ *Sry* males, with predominance of sperm with clear head elongation with or without curvature, and with and without a marked hooked tip (category A and B, 66%), suggesting that presence of additional Y genes within *Sxr* ^a^ facilitates head restructuring. In X^*E*^ *Sxr* ^b^Y*^X^ males the great majority of sperm were scored as elongated but poorly condensed (category G, 74%; this category was specific for this genotype). The tail development in all examined genotypes was normal (see S4 Fig for images of whole testicular sperm from X^*E*,*Z2*^Y*^X^ *Sry* males).

Altogether, our data support that presence of *Zfy2* enables round spermatids to initiate and undergo head morphogenesis and complete tail development. The *Zfy2* in X^*E*,*Z2*^Y*^X^ *Sry* makes this transition much more effectively than *Zfy2/1* in X^*E*^ *Sxr* ^b^Y*^X^ but does not reach the level observed in males with Y gene contribution provided by *Sxr* ^a^. In all genotypes this restructuring does not proceed normally and yields sperm with severely amorphous heads.

### Addition of *Zfy2* to X^*E*^O*Sry* and X^*E*^Y*^X^ *Sry* males increases the success of round spermatids injection (ROSI) and allows for successful ICSI yielding live offspring

In our recent study we have shown that ROSI success with X^*E*^O*Sry* and X^*E*^Y*^X^ *Sry* males was below 10% (9% and 6%, respectively) while with X^*E*^ *Sxr* ^*b*^O and X^*E*^Y*^X^ *Sxr* ^*b*^ males ROSI efficiency increased by ~2–2.5 fold (20% and 16%, respectively), suggesting that a gene/s encoded within *Sxr* ^b^ provides some benefit for assisted reproduction success [5]. Considering the *Zfy2* role in meiotic progression [11] and spermatid elongation (Figs 2 and 3, S3 & S4 Figs) we decided to test whether *Zfy2* is beneficial for germ cell function. When ROSI was performed with round spermatids from X^*E*,*Z2*^O*Sry* and X^*E*,*Z2*^Y*^X^ *Sry* males (Fig 1B, S1 Table) live offspring rate increased, reaching 27% and 43%, respectively (Table 2).

**Table 2:** The results of round spermatid injection (ROSI) and intracytoplasmic sperm injection (ICSI) with germ cells from males with limited Y gene complement.

Male genotype	Y gene contribution	Males yielding progeny	Fetuses % (no)^#^	Implants % (no)^#^	Males yielding progeny	Fetuses % (no)^#^	Implants % (no)^#^
		*ROSI*			*ICSI*		
X^*E*,*Z2*^O*Sry*	*Eif2s3y*, *Sry* & *Zfy2*	2/2	**27** (7/26)	39 (10/26)^a^	no sperm	-	-
X^*E*,*Z2*^Y*^X^ *Sry*	*Eif2s3y*, *Sry* & *Zfy2*	5/5	**43** (48/111)^d^	79 (71/90)^c^	4/4	**23** (18/79)^b^	48 (38/79)^b^
XY	intact Y	4/4	**30** (21/70)^d^	63 (44/70)^c^	3/3	**57** (32/56)	80 (45/56)

^#^ Percentage was calculated from embryos transferred.

Statistical significance (Fisher's Exact Test):

^a^ P<0.05

^b^ P<0.001 vs. respective category in XY control

^c^ P<0.05

^d^ <0.01 vs. ICSI within genotype.

Sperm from X^*E*^ *Sxr* ^*b*^O and X^*E*^ *Sxr* ^*b*^Y*^X^ males were not functional in assisted fertilization, and for X^*E*^ *Sxr* ^b^Y*^X^ males it could be attributed to Y gene deficiency. To test whether *Zfy2* influences ICSI outcome, we performed injections with sperm from an X^*E*,*Z2*^Y*^X^ *Sxr* ^*b*^ male, which had both *Sxr* ^b^ (encoding the *Zfy2/1* fusion gene) and the *Zfy2* transgene (Fig 1B). We had only one male of this genotype available as sperm donor for ICSI, and only 7 embryos were transferred but those yielded 2 live offspring (29%, 2/7). Encouraged by this result we moved on to test sperm from X^*E*,*Z2*^Y*^X^ *Sry* males. Out of 5 males examined, 4 had testicular sperm and yielded live ICSI offspring (Table 2). The efficiency of ICSI with sperm from X^*E*,*Z2*^Y*^X^ *Sry* males was lower than with sperm wild-type XY controls (23% vs. 57%, P<0.001). Because each X^*E*,*Z2*^Y*^X^ *Sry* and XY male provided both round spermatids (ROSI) and sperm (ICSI) for injections, we were able to perform a direct comparison of these two types of germ cells for their ability to participate in successful assisted fertilization. In XY males, as expected, the efficiency of ROSI was lower than ICSI (Table 2, 30% vs. 57%, P<0.01). Interestingly, this pattern was reversed in X^*E*,*Z2*^Y*^X^ *Sry* males, in which ROSI success was significantly better (Table 2, 43% vs. 23%, P<0.01).

**Fig 4:**
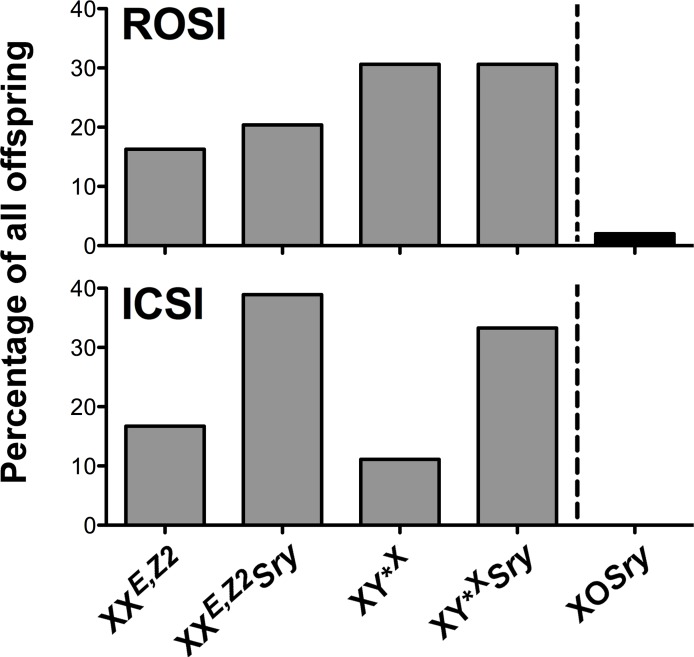
Progeny genotype frequencies. Frequency of the offspring genotypes obtained after assisted reproduction with X^*E*,*Z2*^Y*^X^ *Sry* males. Four predominant genotypes (grey bars) and one rare genotype derived from untypical sex chromosome segregation (black bar on the right side of the dashed line) were observed. These genotypes are expected from X^*E*,*Z2*^Y*^X^ *Sry* males. Number of genotyped progeny was 49 for ROSI and 18 for ICSI. See also S2 Table.

X^*E*,*Z2*^Y*^X^ *Sry* males generate several types of gametes, which consequently lead to several possible progeny genotypes. Genotyping of all progeny obtained after ROSI and ICSI revealed that anticipated offspring types were produced and their frequency met the expectancy, with 4 predominating genotypes accounting for 98.5% of all genotypes and distributed within 16%-31% range, and 1 rare genotype, which originated from atypical segregation of sex chromosomes (Fig 4 and S2 Table).

### *Zfy* expression analysis supports the role of *Zfy2*, and not *Zfy1*, in sperm function

**Fig 5:**
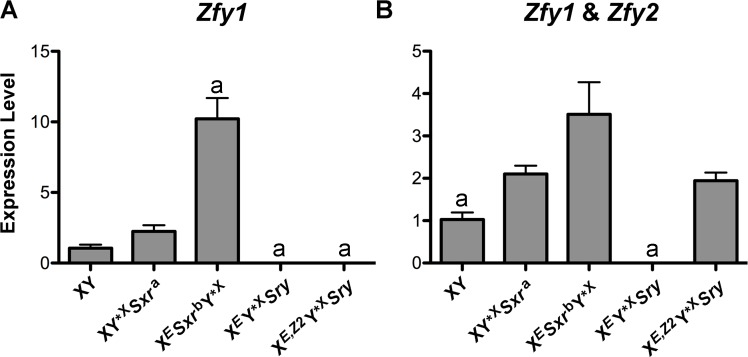
*Zfy* expression. (A) *Zfy1* transcript levels in genotypes of interest (n = 3 per genotype) obtained by real-time PCR. The loading controls were two ubiquitously expressed genes (actin and *Sdha*) and two spermatid-specific genes (*Act* and *Acrv*), and normalization was achieved by geometric averaging of these genes. (B) *Zfy1* and *Zfy2* (global *Zfy*) transcript levels were examined as in A. Values are mean ± SEM. Statistical significance: ^a^ different than all others (except zero to zero values comparison); * P < 0.05. For explanation of genotypes see Fig 1, S1 Table, and text. Primer sequences are shown in S3 Table. The data normalized to individual reference genes are shown in S5 Fig.

To correlate spermiogenic phenotype with *Zfy* expression we performed *Zfy* transcript quantification on whole testes from X^*E*^Y*^X^ *Sry* (no *Zfy*), X^*E*^ *Sxr* ^*b*^Y*^X^ (*Zfy2/1* fusion gene), X^*E*,*Z2*^Y*^X^ *Sry* (*Zfy2* transgene), with XY*^X^ *Sxr* ^*a*^ (endogenous *Zfy2* and *Zfy1* and no NPYq) and XY (intact Y chromosome) serving as controls. Because *Zfy2* and *Zfy1* are very similar (97% and 94% for transcript and amino acid identity, respectively) we failed to design real-time PCR primers that were specific to *Zfy2*. We therefore quantified the expression of *Zfy1*, and *Zfy1* and *Zfy2* combined (global) (Fig 5 and S5 Fig).

As expected, no *Zfy1* transcripts were detected in X^*E*^Y*^X^ *Sry* and X^*E*,*Z2*^Y*^X^ *Sry* males. XY*^X^ *Sxr* ^*a*^ males had ~2-fold higher *Zfy1* levels than XY males but the difference did not reach significance (P = 0.08). This minor increase is likely due to the lack of NPYq genes known to result in the upregulation of sex chromosome genes, including *Zfy* [20]. In X^*E*^ *Sxr* ^*b*^Y*^X^ males *Zfy1* levels were ~10-fold and ~5-fold higher than in XY and XY*^X^ *Sxr* ^*a*^, respectively, representing a combined effect of the NPYq absence and activity of a strong *Cypt*-derived spermatid-specific promoter driving expression of the *Zfy2/1* fusion gene. The global *Zfy* expression was again higher in X^*E*^ *Sxr* ^*b*^Y*^X^ and XY*^X^ *Sxr* ^*a*^ than in XY but the difference was lower in magnitude. *Zfy* global levels in X^*E*,*Z2*^Y*^X^ *Sry* males were similar to those of XY*^X^ *Sxr* ^*a*^, and higher than in XY. When compared to X^*E*^ *Sxr* ^*b*^Y*^X^, *Zfy* global levels in X^*E*,*Z2*^Y*^X^ *Sry* males were ~2-fold lower but the difference did not reach significance (P = 0.1). In X^*E*,*Z2*^Y*^X^ *Sry* males the global levels were reflective exclusively of *Zfy2*, while in X^*E*^ *Sxr* ^*b*^Y*^X^ males primarily of *Zfy1* since the *Zfy2/1* fusion gene encodes *Zfy1* coding region under the control of *Zfy2* promoter [17].

When the spermiogenic phenotype is viewed in the context of *Zfy* expression our data support that it is *Zfy2*, and not *Zfy1* (even if present in abundance), that enables the formation of sperm functional in ICSI.

## Discussion

Y chromosome encoded zinc finger protein genes, *Zfy*, have once been in the center of attention as potential candidates for the testis-determining factors [21–23]. When the fame went to another Y gene, *Sry* [1–3], *Zfy* genes were quickly forgotten and it has taken more than two decades for these genes to re-emerge with newly ascribed spermiogenic roles. *Zfy1* and *Zfy2* were shown to play spermatogenic quality functions during the pachytene stage of meiosis and during MI by triggering the apoptotic elimination of spermatocytes [24,25] and to facilitate the second meiotic division [11]. It has also been shown that a gene/s from *Sxr* ^a^, partially retained in *Sxr* ^b^, is necessary for the initiation of sperm morphogenesis [7] and increases the efficiency of round spermatid injection [5]; *Zfy* genes were proposed as the most likely candidates. Here we tested this assumption by investigating the effects of transgenic *Zfy2* addition into Y chromosome deficient males, which have a postmeiotic arrest at the round spermatid stage. We demonstrated that *Zfy2* is responsible for formation of sperm functional in assisted fertilization.

In our previous study we reported that only two Y chromosome genes, the testis determinant factor *Sry* and the spermatogonial proliferation factor *Eif2s3y*, are sufficient to make a male mouse whose germ cells are functional in assisted fertilization (ROSI) and yield live progeny [5]. When Y chromosome contribution was expanded by substituting *Sry* for *Sxr* ^b^, ROSI efficiency improved, and we speculated that this was due to the presence of the *Zfy2/1* fusion gene, which facilitated the second meiotic division in the testis in the presence of Y*^X^, or in the oocytes after fertilization when the meiotic pairing partner was missing [5]. Spermatid elongation and occasional formation of mature testicular sperm were previously observed in males with *Sxr* ^b^ [5,7] but their function in fertilization has not been tested. Here we have shown that these sperm are not successful in assisted fertilization (ICSI). In X^*E*^ *Sxr* ^*b*^O males the great majority of elongating spermatids are diploid [7] and so are the testicular sperm as shown in this study. The fact that live offspring were obtained with ROSI, and not with ICSI, could therefore be due to the highly condensed nature of the sperm chromatin. In diploid round spermatids from X^*E*^ *Sxr* ^*b*^O males the chromatin is still histone-bound and the homologous chromosomes are presumably still paired as in meiosis II. Secondary spermatocytes, with the same chromosomal state prematurely condense upon injection into oocytes and complete meiosis II along with the maternal chromatin, expelling a polar body-like structure with the haploid complement of paternal DNA [26]. We have proposed that a similar process occurs with the round spermatids from X^*E*^ *Sxr* ^*b*^O males, resulting in normal, diploid zygotes [5]. The diploid spermatozoa, however, cannot have the normal histone component because in order to complete spermiogenesis most of the histones would have had to be replaced by protamines. This sperm chromatin must be completely reorganized, which normally takes one to two hours after ICSI [27]. By this time, the maternal chromatin has already completed meiosis II, and the zygote can no longer support the completion of meiosis II for the paternal DNA. Congruent with this explanation, ICSI should be successful with sperm from X^*E*^ *Sxr* ^*b*^Y*^X^ males, which yielded predominantly diploid zygotes. However, only one offspring was obtained, suggesting that sperm ability to support embryonic and fetal development was highly impaired. We have previously shown that XY*^X^ *Sxr* ^a^ males can be reproduced by ICSI [19]. Thus, one or more Y genes that are present and active in *Sxr* ^a^, and not in *Sxr* ^*b*^, are likely to be responsible for rendering sperm functional. We now show that this gene is *Zfy2*, and that sperm dysfunction in X^*E*^ *Sxr* ^*b*^Y*^X^ males can be overcome with the transgenic addition of *Zfy2*.

Why is it that *Zfy2* renders sperm functional in assisted fertilization while the *Zfy2/1* fusion gene does not? The *Zfy2/1* fusion gene, present within *Sxr* ^b^, encodes a protein that is almost identical to that encoded by *Zfy1* but the transcription is driven by a *Cypt*-derived *Zfy2* specific promoter [17]. Both *Zfy2* and *Zfy2/1* are therefore strongly expressed postmeiotically because both have the *Cypt*-derived promoter, which drives strong expression in spermatids [14]. However, in the case of the *Zfy2/1* fusion gene, the transcript produced is almost identical to that of *Zfy1*. Alternative splicing results in the majority of *Zfy1* transcripts lacking exon 6, which encodes the ZFY protein transactivating domain (TA), while most of the *Zfy2* transcripts retain exon 6 [17]. The protein encoded by *Zfy1* lacking exon 6 is expected to bind but not transactivate target genes and consequently can serve as a competitive inhibitor of full length ZFY proteins. Moreover, the TA domain in ZFY1 protein, when present, is ~5.5-fold less active than that of ZFY2 protein [11]. The *Zfy2/1* fusion gene produces transcripts that are spliced like those of *Zfy1* so that a substantial proportion of them lack the exon 6 encoding the TA domain transcription factor function [17] and when TA domain is present, it is equivalent to that encoded by ZFY1 and therefore less potent. These transcript and protein specific differences explain why *Zfy2/1* in X^*E*^ *Sxr* ^*b*^Y*^X^ males is not sufficient for promoting sperm function, and why addition of *Zfy2* to this genotype rescues this defect.

The *Zfy2* levels in X^*E*,*Z2*^Y*^X^ *Sry* males were ~1.9 fold higher than in XY. It therefore cannot be disregarded that the spermiogenic phenotype results from *Zfy2* overexpression. The *Zfy2* transgene was provided as a single copy *Zfy2* BAC inserted by cassette mediated exchange (CME) into the *Hprt* locus on the X chromosome [24,25]. This transgene, because of its localization on the X chromosome, behaves in the same way as the endogenous gene, i.e. undergoes meiotic sex chromosome silencing (MSCI) [24], and its level of expression should be equivalent to that of the endogenous gene. The overexpression observed in X^*E*,*Z2*^Y*^X^ *Sry* males is likely due to the lack of Y chromosome long arm genes, known to result in global upregulation of several sex chromosome genes, including *Zfy2* [20]. The fact that we see *Zfy* overexpression in XY*^X^ *Sxr* ^a^ males, which have endogenous *Zfy1* and *Zfy2* but lack the Y chromosome long arm, supports this case. To bring the *Zfy2* expression to the physiological level X^*E*,*Z2*^Y*^X^ *Sry* males, we would need to provide the genes from the Y long arm but those would likely influence spermiogenic phenotype. Thus, with the current tools, we cannot test whether *Zfy2* expression at physiological levels would also induce spermatid elongation and promote sperm function. The resolution can come from the analysis of *Zfy2* knockout mice, interpreted in the context of the expression data presented here.

The fact that we obtained ICSI offspring from X^*E*,*Z2*^Y*^X^ *Sry* males represents a significant advancement in establishing a minimum Y complement compatible with successful assisted fertilization. Although we have shown earlier that only two Y genes are sufficient to generate progeny [5], this was achieved with round spermatid injection (ROSI), a method which is considered experimental in human ART due to concerns regarding the safety of injecting immature germ cells and technical difficulties [28]. Intracytoplasmic sperm injection (ICSI), however, is a common procedure in human ART, rendering our mouse data more directly translational.

With the reemergence of *Zfy* genes from the backstage and their recently acknowledged roles during spermatogenesis [5,7,11,17,24,25] (and this study), it will now be important to characterize the mechanism and identify the target genes that these transcription factors regulate. In humans, there is a single *ZFY* gene on the Y chromosome, which is ubiquitously expressed. No mutations of *ZFY* have been described and there is therefore no information concerning its possible contribution to human germ cell development or male fertility. The newly acquired mouse data regarding the role of *Zfy* gene in spermatogenesis may therefore trigger re-evaluation of *ZFY* function in humans.

## Materials and Methods

### Ethics statement

The mice were maintained in accordance with the guidelines of the Laboratory Animal Services at the University of Hawaii and guidelines presented in National Research Council’s (NCR) “Guide for Care and Use of Laboratory Animals” published by Institute for Laboratory Animal Research (ILAR) of the National Academy of Science, Bethesda, MD, 2011. Anesthesia, necessary for performing embryo transfers and semi-castration, was achieved by intraperitoneal injection of Avertin. Euthanasia was achieved by cervival dislocation. MAW has an active protocol for animal handling and treatment procedures (protocol number 06–010), reviewed and approved by local Animal Care and Use Committees annually.

### Chemicals and media

Pregnant mares’ serum gonadotrophin (eCG) and human chorionic gonadotrophin (hCG) were purchased from Calbiochem (San Diego, CA). All other chemicals were obtained from Sigma Chemical Co. (St Louis, MO) unless otherwise stated. Sperm and oocyte collection and subsequent manipulation, including microinjections were done in HEPES-buffered CZB medium (HEPES-CZB) [29]. Culture of injected oocytes and embryos was done in CZB medium [30].

### Animals

The mice of interest in this study were mice with limited Y gene complement (Fig 1, S1 Table):

1. X^*Eif2s3y*^O*Sry* (abbreviated as X^*E*^O*Sry*) are males carrying an autosomally-encoded transgene of testis determinant *Sry* [31] and the X chromosome-located transgene encoding spermatogonial proliferation factor *Eif2s3y* [32]. These mice have only one sex chromosome (hence the designation XO).
2. X^*Eif2s3y*^Y*^X^ *Sry* (abbreviated as X^*E*^Y*^X^ *Sry*) males have the same Y gene complement as X^*E*^O*Sry* but carry a minute X chromosome derivative (Y*^X^) with a complete pseudoautosomal region (PAR) but lacking most of the other X genes [33].
3. X^*Eif2s3y*^ *Sxr* ^b^O (abbreviated as X^*E*^ *Sxr* ^b^O) males have the X chromosome carrying an *Eif2s3y* transgene [32] together with Tp(Y)1Ct^*Sxr-b*^, a *Sxr* ^a^ derivative with a 1.3 Mb deletion that has removed the majority of the Yp gene complement and created a *Zfy2/1* fusion gene [34,35].
4. X^*Eif2s3y*^ *Sxr* ^b^Y*^X^ (abbreviated as X^*E*^ *Sxr* ^b^Y*^X^) have the same Y gene complement as X^*E*^ *Sxr* ^b^O but carry also Y*^X^.
5. XY* ^X^ *Sxr* ^*a*^ have a single X chromosome and Tp(Y)1Ct^*Sxr-a*^ [36] attached distal to the Y*^X^ PAR.
6. Mice with *Zfy2* transgene. The *Zfy2* transgene (abbreviated as ^*Z2*^) was added to the genotypes described in 1, 2 & 4. It was provided as a single copy *Zfy2* BAC inserted by cassette mediated exchange (CME) into the *Hprt* locus on the X chromosome [24,25].

The X^*E*^O*Sry* and X^*E*^Y*^X^ *Sry* males were produced ‘in house’ by breeding X^*Paf*^O or X^*Paf*^Y*^X^ females [37] carrying the X-linked coat marker *Patchy-fur* [38] and X^*Eif2s3y*^Y^*Tdym1*^ *Sry* males that have the X chromosome carrying an *Eif2s3y* transgene [32] and a Y chromosome with an 11-kb deletion removing the testis determinant *Sry* (*dl1Rlb*) [1,39], complemented by an autosomally-located *Sry* transgene [Tg(Sry)2Ei] [31]. The X^*E*^ *Sxr* ^b^O and X^*E*^ *Sxr* ^b^Y*^X^ males were produced ‘in house’ by breeding X^*Paf*^O or X^*Paf*^Y*^X^ females described above and X^*Eif2s3y*^Y*Sxr* ^b^ males that have the X chromosome carrying an *Eif2s3y* transgene [32] and a Y-chromosome that has Tp(Y)1Ct^*Sxr-b*^ sex reversal factor [40,41] attached distal to its PAR region. The XY*^X^ *Sxr* ^a^ males were produced by ICSI with sperm from males of the same genotype and oocytes from wild-type females. Males transgenic for *Zfy2* (X^*E*,*Z2*^O*Sry*, X^*E*,*Z2*^Y*^X^ *Sry* and X^*E*,*Z2*^ *Sxr* ^b^Y*^X^) were produced as described above but with the father carrying X^*E*,*Z2*^ rather than X^*E*^.

The crosses utilized in production of mice with limited Y gene complement yield a variety of progeny genotypes. The males of interest were identified among the progeny by genotyping for X and Y chromosome markers, scoring fur appearance, and evaluation of testes size. All mice were on MF1 genetic background, except for XY* ^X^ *Sxr* ^*a*^ which was C57BL/6. XY^RIII^ MF1 were used as wild-type controls; Y^RIII^ chromosome is the strain of Y chromosome from which *Sxr* ^a^ and *Sxr* ^b^ derive.

For assisted reproduction, six-to-twelve week-old B6D2F1 (C57BL/6 x DBA/2) females (NCI, Raleigh, NC) were used as oocyte donors and CD-1 (Charles River, Wilmington, MA) or Swiss Webster (NCI, Raleigh, NC) mice were used as vasectomized males and surrogate/foster females for embryo transfer.

The mice were fed ad libitum with a standard diet and maintained in a temperature and light-controlled room (22°C, 14h light/10h dark), in accordance with the guidelines of the Laboratory Animal Services at the University of Hawaii and guidelines presented in National Research Council’s (NCR) “Guide for Care and Use of Laboratory Animals” published by Institute for Laboratory Animal Research (ILAR) of the National Academy of Science, Bethesda, MD, 2011. The protocol for animal handling and treatment procedures was reviewed and approved by local Animal Care and Use Committees. Anesthesia, necessary for performing embryo transfers and semi-castration, was achieved by intraperitoneal injection of Avertin. Euthanasia was achieved by cervival dislocation. MAW has an active protocol for animal handling and treatment procedures (protocol number 06–010), reviewed and approved by local Animal Care and Use Committees annually.

### Histology analysis

For histology analysis, part of the testes were fixed in Bouin overnight and then stored in 70% ethanol prior to embedding in paraffin wax, sectioning at 5 μm, and staining with hematoxylin-eosin (H&E) and Periodic acid Schiff and hematoxylin (PAS-H). The stages of seminiferous tubules were identified based on the composition of cells near the basal membrane according to the method described by Ahmed [42], and as described by us before [5]. This was necessary because of meiotic and post-meiotic arrests present in males with limited Y gene complement, which prevented staging based on the changes of acrosome and nuclear morphology of spermatids.

### Sperm morphology analysis

Sperm morphology was examined on surface spreads of spermatogenic cells prepared from frozen testis tissue as described earlier [25]. The images of sperm were captured at 1000x magnification.

### Round spermatid injection (ROSI) and intracytoplasmic sperm injection (ICSI)

Injections with testicular cells were performed as described before [5,19]. Testes were collected twice from each male following initial semi-castration, and used for preparation of testicular cell suspensions for injections. The metaphase II (MII) oocytes for ROSI were collected from superovulated (5 iu eCG and 5 iu hCG given 48 hrs apart) female mice and incubated at 37°C, 5% CO_2_ until injection. Testicular sperm suspension was diluted with HEPES-CZB containing 1% (w/v) polyvinyl pyrrolidone (PVP) on the injection dish. Spermatids were injected individually into the oocytes. The total duration of ROSI was no longer than 1 hour. The oocytes were activated shortly after injection by incubation in Ca^2+^-free CZB medium supplemented with 2.5 mM SrCl_2_ at 37°C, 5% CO_2_ for 4 hrs, after which time they were transferred into standard CZB medium for subsequent culture. At ~6–8 hrs after injection the oocytes were assessed for polar body extrusion and pronuclei development. Normally fertilized oocytes exhibiting two pronuclei (PN) and extruded second polar body (PBII) were allowed to cleave and were subjected to embryo transfer. Surrogate mothers were subjected to caesarian section on day 18 of pregnancy to allow for scoring of the numbers of fetuses and implantation sites.

### Zygote chromosome analysis

Chromosome preparation and analysis were performed as previously described [8]. In wild-type males the spermatid used for injection is considered chromosomally normal when resulting zygote contains 40 normal metaphase chromosomes, 20 maternal and 20 paternal. Males with limited Y gene complement either lack the Y chromosome or carry a minute Y*^X^ chromosome variant. Thus, for these genotypes lack of one chromosome in the paternal chromosome complement and the presence of one small variant were considered normal.

### Progeny genotyping

Offspring produced with ICSI and ROSI were genotyped by PCR to identify presence of *Eif2s3y*, *Zfy2*, and *Sry* transgenes. Presence of Y*^X^ was recognized by copy number assessment. Genomic DNA was isolated from mouse tails using phenol chloroform extraction and ethanol precipitation. DNA was used to amplify single copies of X-linked *Prdx4* (absent in Y*^X^) and *Amelx* (present in Y*^X^), and *Atr* (on chromosome 9) for normalization using *Power* SYBR Green PCR Master Mix on a Quant Studio 12K Flex machine (Applied Biosystems). The following conditions were used: 95^°^C for 10 min, followed by 40 cycles of 95^°^C for 10 sec and 60^°^C for 60 sec. Two PCR reactions were used to detect the presence of Y*^X^ and the number of X-chromosomes. An 82-bp *Prdx4* fragment were amplified using primers *Prdx4-*F and *Prdx4*-R and a 162-bp *Amelx* fragment with primers *Amelx*-F and *Amelx*-R. All samples were tested in quadruplicate per assay using XO samples as a reference control. Copy number estimation for each gene was calculated with the ΔΔCt method. Briefly, ΔCt values were calculated as difference between tested gene and *Atr*. ΔΔCt values were calculated by subtracting ΔCt of tested genes from the reference samples. The copy numbers were calculated by raising 2 to the power of ΔΔCt (2^ΔΔCt^). The genotypes were inferred from the copies of each target gene: XO, 1 *Prdx4* + 1 *Amelx;* XY*^X^, 1 *Prdx4* + 2 *Amelx*; XX, 2 *Prdx4* + 2 *Amelx*; XXY*^X^, 2 *Prdx4* + 3 *Amelx*. Primer sequences are shown in S3 Table.

### Real-time RT-PCR

For real-time reverse transcriptase polymerase chain reaction (RT-PCR), total testis RNA was extracted using Trizol and DNaseI treatment (Ambion, Austin, TX,USA), and purified using an RNeasy kit (Qiagen, Valencia, CA, USA). Reverse transcription of polyadenylated RNA was performed with Superscript Reverse Transcriptase III, according to the manufacturer’s guidelines (Invitrogen, Carlsbad, CA, USA). Real-time PCR was performed using SYBR Green PCR Master mix on an ABI QuantStudio 12K Flex machine (Applied Biosystems, Carlsbad, CA, USA). PCR reactions were incubated at 95°C for 10 min followed by 40 PCR cycles (10 s at 95°C and 60 s at 60°C). For analysis of *Zfy* expression, two types of PCR reactions were performed: (1) ‘*Zfy1*’ amplifying only *Zfy1* transcripts and (2) ‘*Zfy* Global‴ amplifying both *Zfy1* and *Zfy2*. Three mice per genotype were analyzed, all reactions were carried out in quadruplicates per assay, and four different loading controls, two ubiquitously expressed genes (actin and *Sdha*) and two spermatid-specific genes (*Act* and *Acrv*) were used. DCt value for each individual sample was calculated by subtracting either the average Ct or geometric mean of loading control(s) from the average Ct of a tested gene. DDCt value was calculated by subtracting the DCt of each tested male from the average DCt of wild-type XY males, which served as references. The data were expressed as a fold value of expression level.

### Statistical analyses

Fisher's Exact Test was used to assess the differences between the genotypes for ROSI and ICSI and zygotic chromosome analysis data. Student t-test was used for gene expression and histology analyses.

## Supporting Information

S1 Fig Testicular sperm from X^*E*^ *Sxr* ^b^Y*^X^ males. Examples of sperm found in live testicular sperm suspension from two different X^*E*^ *Sxr* ^b^Y*^X^ males. Arrows show sperm with tails and arrowheads separated sperm heads, both of which could be found in testis cell suspension. Also visible is a pipette used to transfer sperm. Scale = 10 μm. This figure is related to Table 1.
(TIF)

S2 Fig Exemplary zygotic chromosome spreads. Intracytoplasmic injection of sperm from X^*E*^Sxr^*b*^Y*^X^ males yielded zygotes containing either ~40 chromosomes (left panel, diploid) or ~60 chromosomes (right panel, triploid) indicating that injected sperm varied in respect to their chromosome number. Lower number of chromosomes (38 instead of expected 40 and 56 instead of expected 60) is either because of chromosome loss during preparation or because of chromosome aberrations (fragments, breaks, translocations), which account for the lower number countable chromosomes overall. cf = chromosome fragments. Bar = 50 μm. This figure is related to Table 1.
(TIF)

S3 Fig Histology analysis. Exemplary tubules of stage VII-VIII testis sections from X^*E*^Y*^X^ *Sry*, X^*E*^ *Sxr* ^b^Y*^X^, X^*E*,*Z2*^Y*^X^ *Sry*, and XY males, with spermatids at step 7–8 of development shown in insets. Tubule stages are shown in Roman numerals and steps of spermatid development (St) in Arabic numerals. Bar = 50 μm; insets = x3 magnification. This figure is related to Fig 2
(TIF)

S4 Fig Sperm from X^*E*,*Z2*^Y*^X^ *Sry* males. (A) Examples of sperm found in live epididymal cell suspension from X^*E*,*Z2*^Y*^X^ *Sry* males. Top and bottom panels represent examples of sperm from two different males. Round cells likely represent shed testicular germ cells. (B) Examples of testicular sperm from X^*E*,*Z2*^Y*^X^ *Sry* males identified on silver stained spreads of testicular cells. Scale = 10 μm.
(TIF)

S5 Fig *Zfy* expression data normalized to individual reference genes. *Zfy1* and *Zfy1* and *Zfy2* (global *Zfy*) transcript levels transcript levels in genotypes of interest (n = 3 per genotype) obtained by real-time PCR. The same samples were run independently with four different loading controls, two ubiquitously expressed genes (actin and *Sdha*) and two spermatid-specific genes (*Act* and *Acrv*). Values are mean ± SEM. Statistical significance: ^a^ different than all others (except zero to zero values comparison); * P < 0.05. Primer sequences are shown in S3 Table. This figure is related to Fig 5.
(TIF)

S1 Table The summary of phenotypic features in males with limited Y gene complement. This table is related all figures and tables, and text.
(DOCX)

S2 Table The genotypes of offspring obtained after ICSI and ROSI with germ cells from X^*E*,*Z2*^Y*^X^ *Sry* males. This table is related to Fig 5.
(DOCX)

S3 Table Primers for genotyping and expression analyses. This table is related to Figs 4, 5, S5 and S2 Table.
(DOCX)

S1 Text Supplemental references (DOCX)
