# Supplementary figures and images for: Mouse Y-Encoded Transcription Factor Zfy2 Is Essential for Sperm Formation and Function in Assisted Fertilization
Source: PLoS Genet. 2015 Dec 31;11(12):e1005476. doi: 10.1371/journal.pgen.1005476 (PMC4697804; doi:10.1371/journal.pgen.1005476)

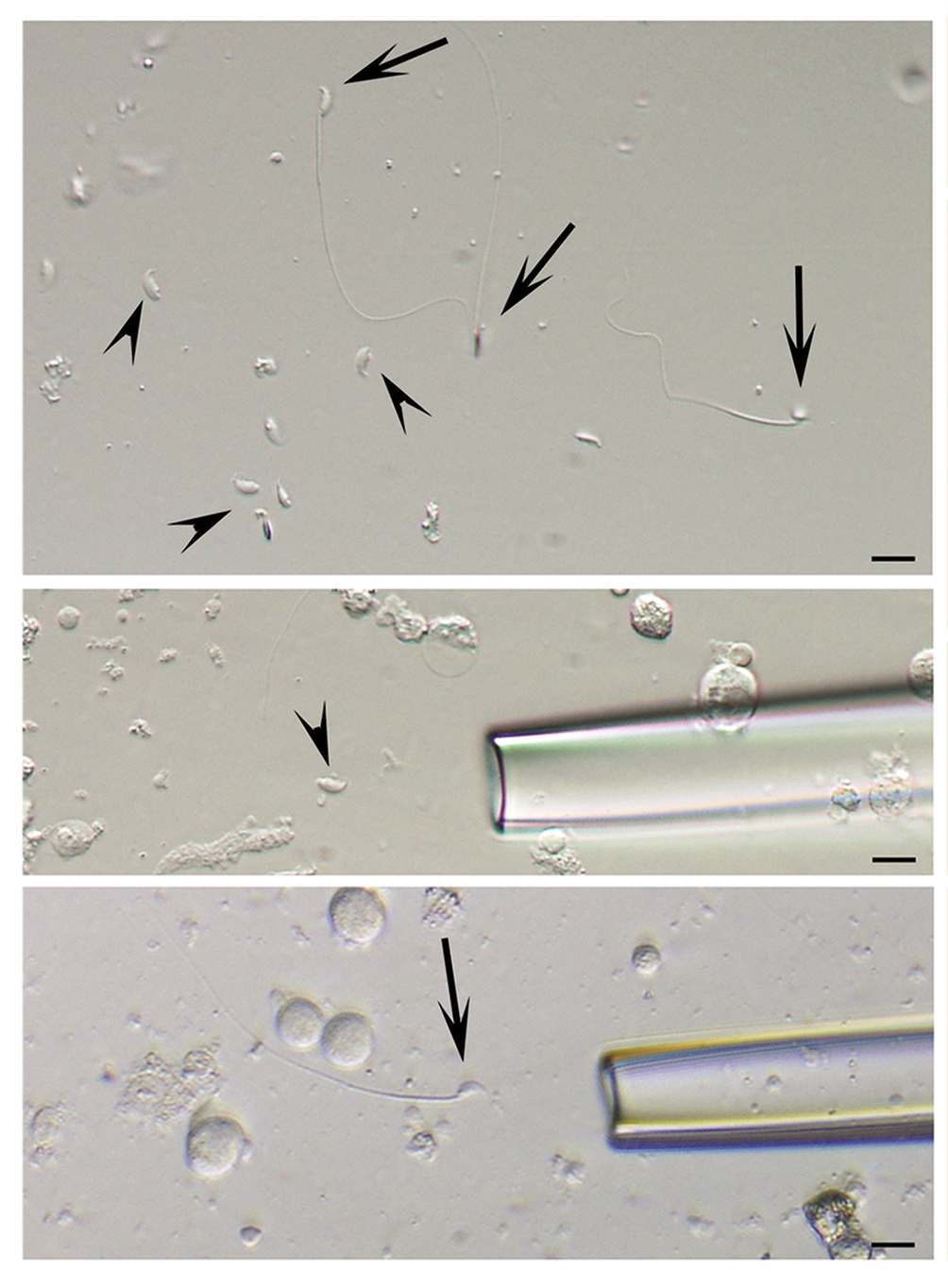

Supplement: S1 Fig — Examples of sperm found in live testicular sperm suspension from two different XE Sxr bY*X males. Arrows show sperm with tails and arrowheads separated sperm heads, both of which could be found in testis cell suspension. Also visible is a pipette used to transfer sperm. Scale = 10 μm. This figure is related to Table 1. (TIF) [file pgen.1005476.s001.tif]

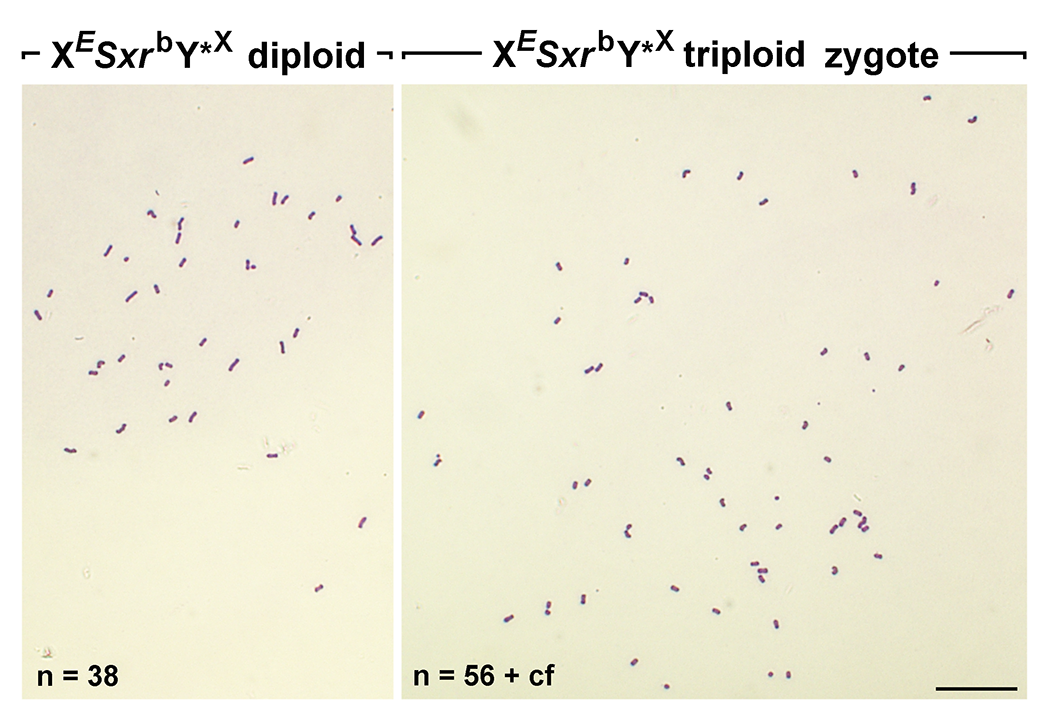

Supplement: S2 Fig — Intracytoplasmic injection of sperm from XESxrbY*X males yielded zygotes containing either ~40 chromosomes (left panel, diploid) or ~60 chromosomes (right panel, triploid) indicating that injected sperm varied in respect to their chromosome number. Lower number of chromosomes (38 instead of expected 40 and 56 instead of expected 60) is either because of chromosome loss during preparation or because of chromosome aberrations (fragments, breaks, translocations), which account for the lower number countable chromosomes overall. cf = chromosome fragments. Bar = 50 μm. This figure is related to Table 1. (TIF) [file pgen.1005476.s002.tif]

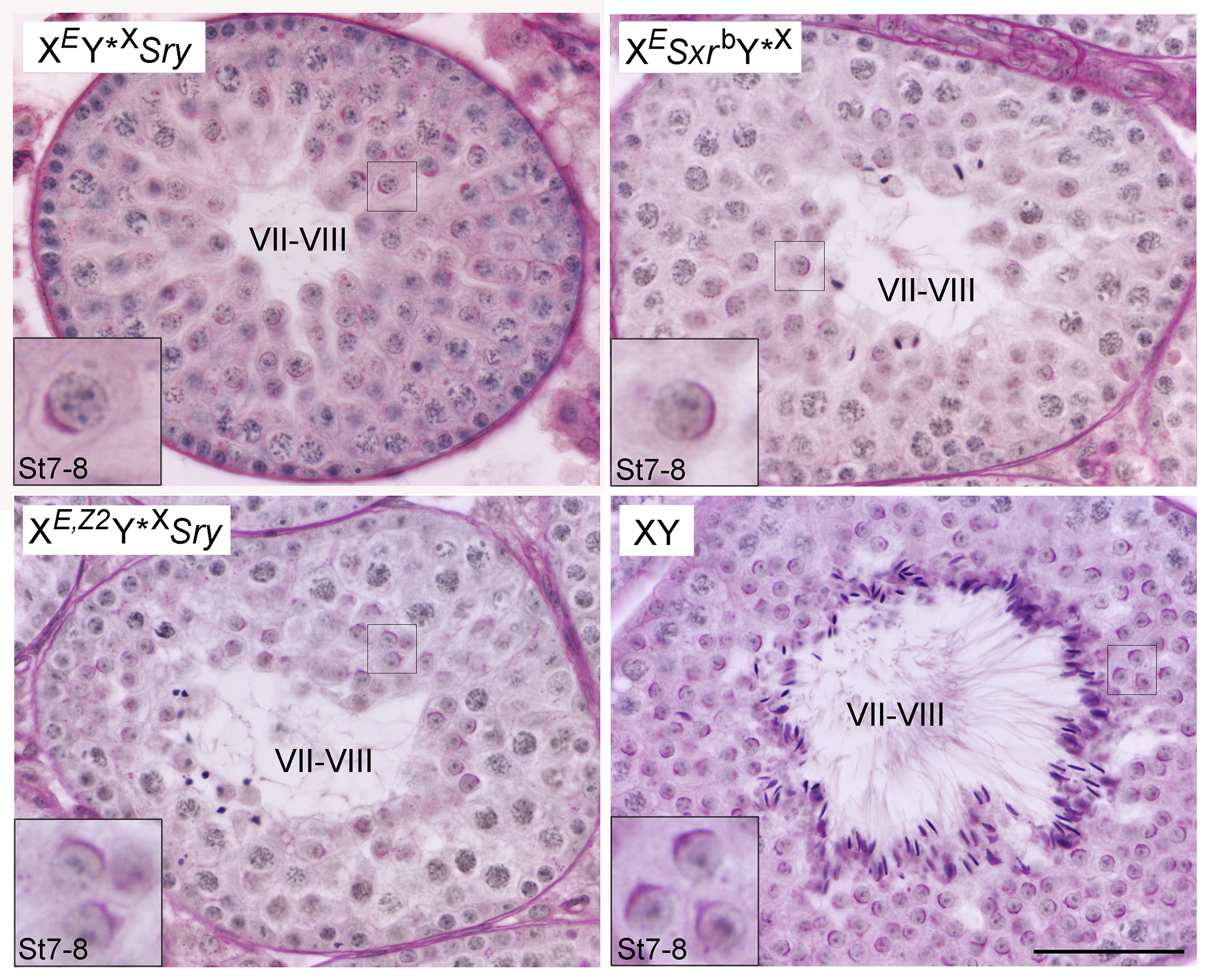

Supplement: S3 Fig — Exemplary tubules of stage VII-VIII testis sections from XEY*X Sry, XE Sxr bY*X, XE,Z2Y*X Sry, and XY males, with spermatids at step 7–8 of development shown in insets. Tubule stages are shown in Roman numerals and steps of spermatid development (St) in Arabic numerals. Bar = 50 μm; insets = x3 magnification. This figure is related to Fig 2 (TIF) [file pgen.1005476.s003.tif]

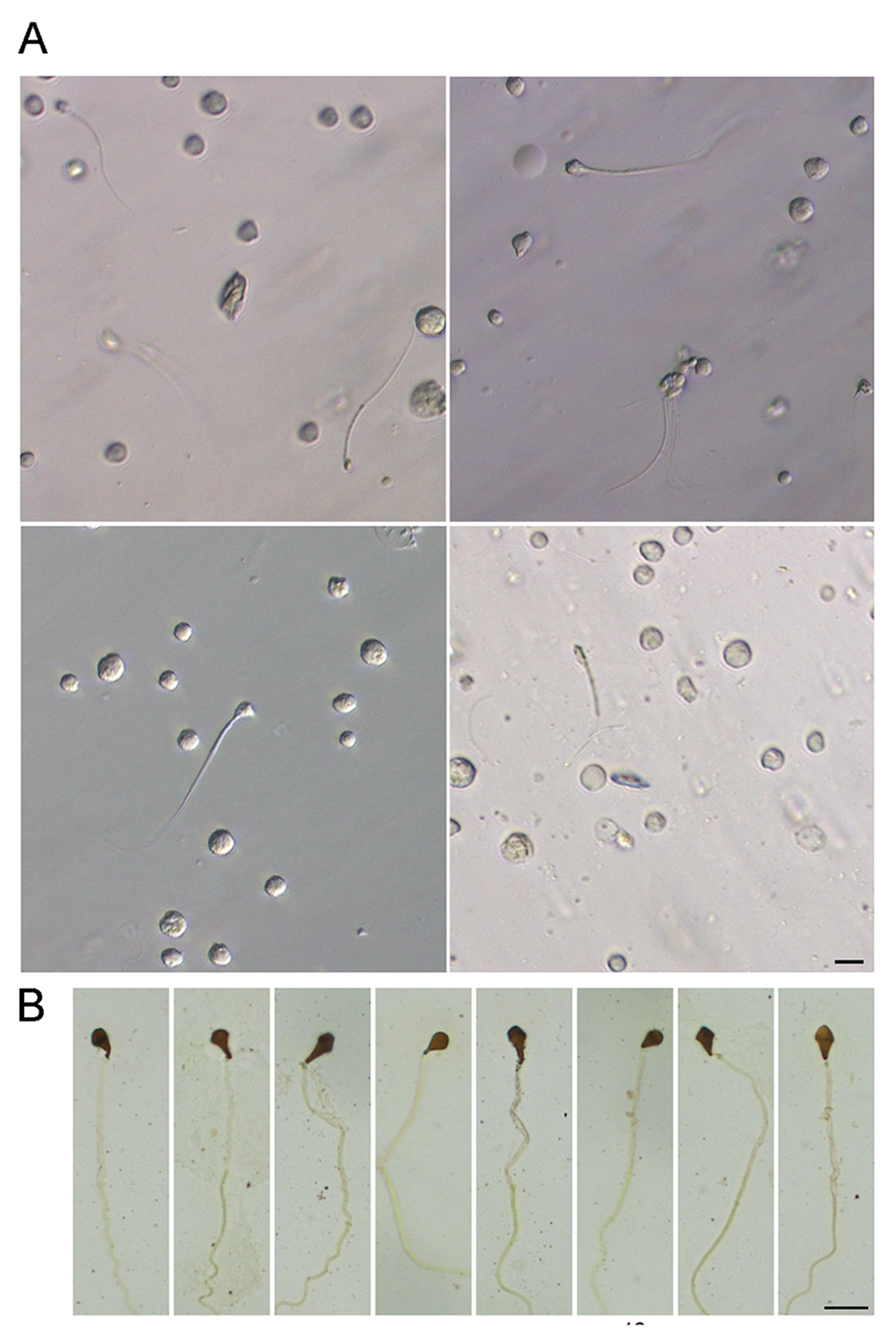

Supplement: S4 Fig — (A) Examples of sperm found in live epididymal cell suspension from XE,Z2Y*X Sry males. Top and bottom panels represent examples of sperm from two different males. Round cells likely represent shed testicular germ cells. (B) Examples of testicular sperm from XE,Z2Y*X Sry males identified on silver stained spreads of testicular cells. Scale = 10 μm. (TIF) [file pgen.1005476.s004.tif]

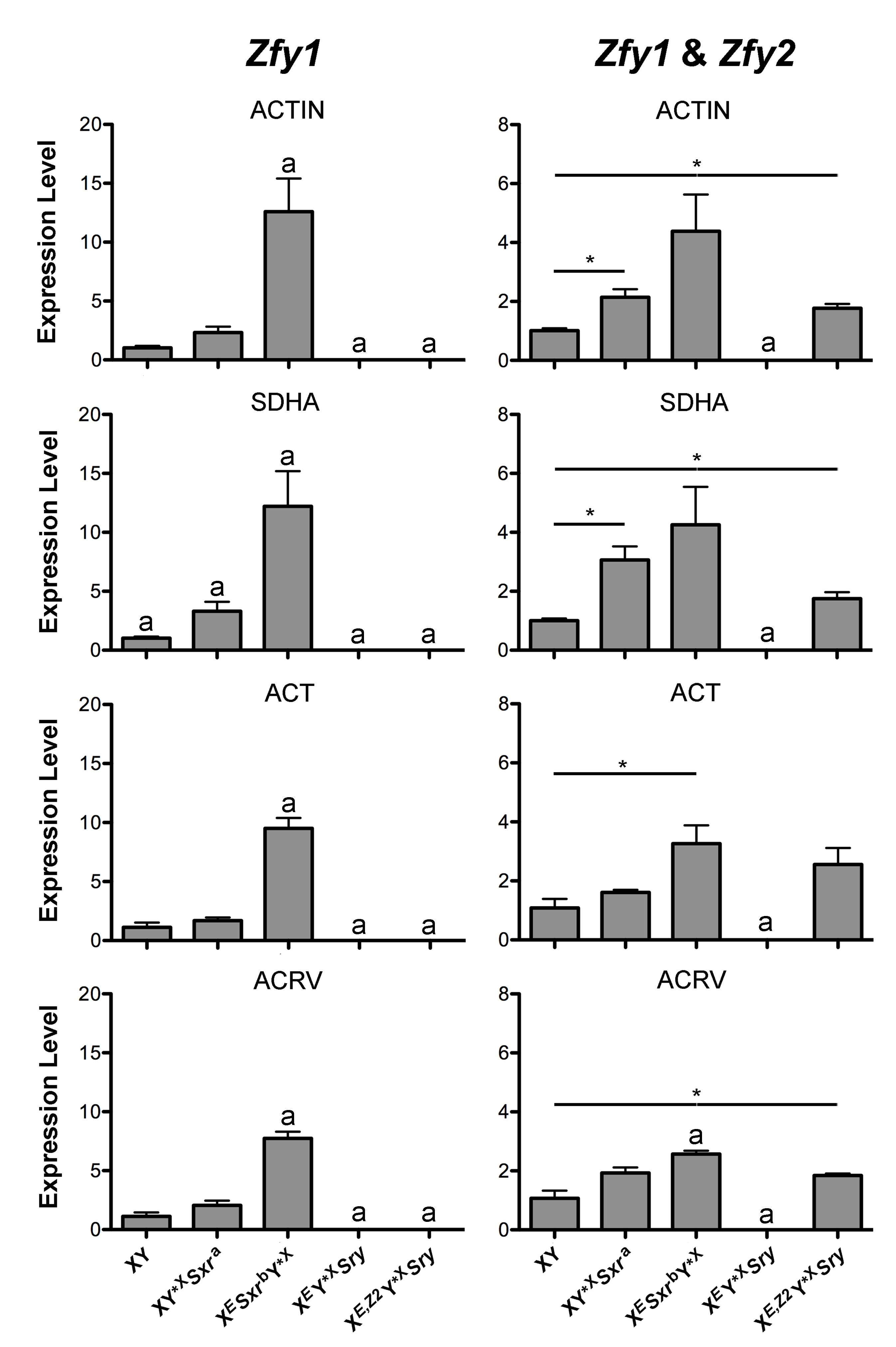

Supplement: S5 Fig — Zfy1 and Zfy1 and Zfy2 (global Zfy) transcript levels transcript levels in genotypes of interest (n = 3 per genotype) obtained by real-time PCR. The same samples were run independently with four different loading controls, two ubiquitously expressed genes (actin and Sdha) and two spermatid-specific genes (Act and Acrv). Values are mean ± SEM. Statistical significance: a different than all others (except zero to zero values comparison); * P < 0.05. Primer sequences are shown in S3 Table. This figure is related to Fig 5. (TIF) [file pgen.1005476.s005.tif]
