# Supplementary material for: Mouse Y-Encoded Transcription Factor Zfy2 Is Essential for Sperm Formation and Function in Assisted Fertilization
Source: PLoS Genet. 2015 Dec 31;11(12):e1005476. doi: 10.1371/journal.pgen.1005476 (PMC4697804; doi:10.1371/journal.pgen.1005476)
Supplement: S1 Table — This table is related all figures and tables, and text. (DOCX) [file pgen.1005476.s006.docx]

S1 Table. The summary of phenotypic features in males with limited Y gene complement.

| Male genotype | Y gene contribution | Sex chromosome pairing | Haploid spermatids | Diploid ROSI zygotes | ROSI offspring | Spermatid elongation | Sperm | Diploid ICSI zygotes | ICSI offspring |
| --- | --- | --- | --- | --- | --- | --- | --- | --- | --- |
| X*^E^*O*Sry* | *Eif2s3y* & *Sry* | no | low | low | low | no | no | n/a | n/a |
| X*^E,Z2^*O*Sry* | *Eif2s3y*, *Sry*,***Zfy2*** | no | low | n/t | high | no | no | n/a | n/a |
| X*^E^*Y*^X^*Sry* | *Eif2s3y* & *Sry* | yes | low | low | low | no | no | n/a | n/a |
| X*^E,Z2^*Y*^X^*Sry* | *Eif2s3y*, *Sry*,***Zfy2*** | yes | high | n/t | high | yes | yes | n/t | yes |
| X*^E^Sxr*^b^O | *Eif2s3y* & *Sxr*^b^ | no | low | high | high | yes | yes | no | no |
| X*^E^Sxr*^b^Y*^X^ | *Eif2s3y* & *Sxr*^b^ | yes | high | high | high | yes | yes | yes | no* |
| X*^E,Z2^Sxr*^b^Y*^X^ | *Eif2s3y*, *Sxr*^b^,***Zfy2*** | yes | n/t | high | n/t | yes | yes | n/t | yes |
| XY*^X^*Sxr*^a^ | *Sxr*^a^ | yes | n/t | n/t | high | yes | yes | high | yes |
| XY^RIII^ | intact Y | yes | high | high | high | yes | yes | high | yes |

*Sxr*^b^: *Prrsly*, *Teyorf1*, *Sry, Zfy2/1*, *H2al2y*, *Rbmy* cluster (7 copies) (See Fig. 1)

*Sxr*^a^: All NPYp genes with *Rbmy* cluster reduced to 7 copies (See Fig. 1)

n/a = not applicable; n/t = not tested

* Although one pup was produced, the overall efficiency was so low (1.2%) that we consider it as 'no ICSI offspring'.

The information shown in this table derives from this study as well as previously published papers [1-5]
