# Supplementary material for: Mouse Y-Encoded Transcription Factor Zfy2 Is Essential for Sperm Formation and Function in Assisted Fertilization
Source: PLoS Genet. 2015 Dec 31;11(12):e1005476. doi: 10.1371/journal.pgen.1005476 (PMC4697804; doi:10.1371/journal.pgen.1005476)
Supplement: S2 Table — This table is related to Fig 5. (DOCX) [file pgen.1005476.s007.docx]

**S2 Table.** The genotypes of offspring obtained after ICSI and ROSI with germ cells from X*^E,Z2^*Y*^X^*Sry* males.

| Male ID | Total pups | No (%) pups of the genotype^#^: | | | | |
| --- | --- | --- | --- | --- | --- | --- |
|  |  | XX*^E,Z2^* | XX*^E,Z2^Sry* | XY*^X^ | XY*^X^*Sry* | XO*Sry* |
| M1 | 20 | 5 (25) | 3 (15) | 6 (30) | 5 (25) | 1 (5) |
| M5 | 14 | 2 (14.3) | 3 (21.4) | 6 (42.9) | 3 (21.4) | 0 |
| M7 | 17 | 4 (23.5) | 4 (23.5) | 2 (11.8) | 7 (41.2) | 0 |
| M8 | 10 | 0 | 4 (40) | 3 (30) | 3 (30) | 0 |
| M10 | 6 | 0 | 3 (50) | 0 | 3 (50) | 0 |
| **All** | **67** | **11 (16.4)** | **17 (25.4)** | **17 (25.4)** | **21 (31.3)** | **1 (1.5)** |

^#^ The genotype in shaded column is rare and originate from untypical segregation of sex chromosomes. X chromosome in blue font represents X of oocyte origin. See Fig. 4 for distinction between ROSI and ICSI progeny.
