# Supplementary material for: Mouse Y-Encoded Transcription Factor Zfy2 Is Essential for Sperm Formation and Function in Assisted Fertilization
Source: PLoS Genet. 2015 Dec 31;11(12):e1005476. doi: 10.1371/journal.pgen.1005476 (PMC4697804; doi:10.1371/journal.pgen.1005476)
Supplement: S3 Table — This table is related to Figs 4, 5, S5 and S2 Table. (DOCX) [file pgen.1005476.s008.docx]

**S3 Table.** Primers for genotyping and expression analyses

| Gene | Primer ID | Primer sequence | Amplicon size | Reference |
| --- | --- | --- | --- | --- |
| *qPCR primers for sex chromosome copy number estimation* | | | | |
| *Prdx* | *Prdx4*‑F | CATGATATCCACTGAAAGCTAC | 82 bp | [3] |
|  | *Prdx4*‑R | GAGACAGTGTATCTATCCCTG |  |  |
| *Amelx* | *Amelx*‑F | GTTGGGTTGGAGTCATGGAG | 162 bp | [3] |
|  | *Amelx*‑R | GGCTGCACCACCAAATCATC |  |  |
| *Atr* | *Atr-*WT L1 | GGGATGTTTACAGCCAGCTC | 143 bp | [6] |
|  | *Atr-*WT R1 | AGCCGATTTGCCACAGTAAC |  |  |
| *Standard genotyping PCR primers* | | | | |
| *Myog* | *Om1a* | TTACGTCCATCGTGGACAGCAT | 246 bp | [7] |
|  | *Om1b* | TGGGCTGGGTGTTAGCCTTAT |  |  |
| *Zfy* | *Zfyp1* | AAGATAAGCTTACATAATCACATGGA | 600 bp | [8] |
|  | *Zfyp2* | CCTATGAAATCCTTTGCTGCACATGT |  |  |
| *Sxr*^b^ | *o3452* | GTTAATGAATTAGGGATGGG | 852 bp | [9] |
|  | *o3072* | GTATTAAGTCTTAAAGACATGG |  |  |
| *Ssty* | *Ymtfp1* | CTGGAGCTCTACAGTGATGA | 342 bp | [10] |
|  | *Ymtrp1* | CAGTTACCAATCAACACATCAC |  |  |
| *Sry* (Y*^Tdym1^*) | *Muty3* | GTGTCTCAAAGCCTGCTCTTC | 204 bp | [11] |
|  | *Mutyrp1* | CATGTACTGCTAGCAGCTATC |  |  |
| *Eif2s3y* tg | *EyTspF8* | CAGCTCTGAGGGTGGGTAGTAG | 782 bp | Current paper |
|  | *EyTspR8* | TGACCATGATTACGCCAAGCTAT |  |  |
| *Zfy2* tg | *Zfy2-F* | ttccatttgtcacgtcctgc | 600 bp | Current paper |
|  | *Zfy2-R* | gagtattctatagtgtcacc |  |  |
| *Real-time PCR primers for Zfy expression quantification* | | | | |
| *Zfy Global* | *ZfyGlobalF* | ACAATGCATACAGAAAAAG | 95 bp | Current paper |
|  | *ZfyGlobalR* | AAAAGGTGGTGATTCAAT |  |  |
| *Zfy1* | *Zfy1-spF* | TGGGACTTTGTGTACTCA | 117 bp | Current paper |
|  | *ZfyGlobalR* | AAAAGGTGGTGATTCAAT |  |  |
| *Acrv* | *Acrv-1F* | TGAGTACACCACTTCCAAGCA | 60 bp | [12] |
|  | *Acrv-1R* | AAGCACATGTGTGGCAATTT |  |  |
| *Act* | *Act-F* | CAGCCTGCACCAAACCCA | 117 bp | [13] |
|  | *Act-R* | CACCCACCAAGGAGACCGA |  |  |
| *Actin* | *Actin-F* | GGCACCACACCTTCTACAATG | 352 bp | [14] |
|  | *Actin-R* | GTGGTGGTGAAGCTGTAGCC |  |  |
| *Sadha* | *Sadha-F* | TGTTCAGTTCCACCCCAC | 66 bp | [15] |
|  | *Sadha-R* | TCTCCACGACACCCTTCTGT |  |  |

*Amplified product was digested with 10U of *RsaI* for at least 1h at 37ºC; a 224bp band confirms the presence of *Sxr*^b^.
