# Supplementary material for: Mouse Y-Encoded Transcription Factor Zfy2 Is Essential for Sperm Formation and Function in Assisted Fertilization
Source: PLoS Genet. 2015 Dec 31;11(12):e1005476. doi: 10.1371/journal.pgen.1005476 (PMC4697804; doi:10.1371/journal.pgen.1005476)
Supplement: S1 Text — (DOCX) [file pgen.1005476.s009.docx]

**S1 Text. Supplemental References**

1. Vernet N, Mahadevaiah SK, Ellis PJ, de Rooij DG, Burgoyne PS (2012) Spermatid development in XO male mice with varying Y chromosome short-arm gene content: evidence for a Y gene controlling the initiation of sperm morphogenesis. Reproduction 144: 433-445.

2. Vernet N, Mahadevaiah SK, Ojarikre OA, Longepied G, Prosser HM, et al. (2011) The Y-encoded gene zfy2 acts to remove cells with unpaired chromosomes at the first meiotic metaphase in male mice. Curr Biol 21: 787-793.

3. Vernet N, Mahadevaiah SK, Yamauchi Y, Decarpentrie F, Mitchell MJ, et al. (2014) Mouse Y-linked Zfy1 and Zfy2 are expressed during the male-specific interphase between meiosis I and meiosis II and promote the 2nd meiotic division. PLoS Genet 10: e1004444.

4. Yamauchi Y, Riel JM, Stoytcheva Z, Ward MA (2014) Two Y genes can replace the entire Y chromosome for assisted reproduction in the mouse. Science 343: 69-72.

5. Yamauchi Y, Riel JM, Wong SJ, Ojarikre OA, Burgoyne PS, et al. (2009) Live offspring from mice lacking the Y chromosome long arm gene complement. Biol Reprod 81: 353-361.

6. Royo H, Polikiewicz G, Mahadevaiah SK, Prosser H, Mitchell M, et al. (2010) Evidence that meiotic sex chromosome inactivation is essential for male fertility. Curr Biol 20: 2117-2123.

7. Wright WE, Sassoon DA, Lin VK (1989) Myogenin, a factor regulating myogenesis, has a domain homologous to MyoD. Cell 56: 607-617.

8. King TR, Christianson GJ, Mitchell MJ, Bishop CE, Scott D, et al. (1994) Deletion mapping by immunoselection against the H-Y histocompatibility antigen further resolves the Sxra region of the mouse Y chromosome and reveals complexity of the Hya locus. Genomics 24: 159-168.

9. Decarpentrie F, Vernet N, Mahadevaiah SK, Longepied G, Streichemberger E, et al. (2012) Human and mouse ZFY genes produce a conserved testis-specific transcript encoding a zinc finger protein with a short acidic domain and modified transactivation potential. Hum Mol Genet 21: 2631-2645.

10. Turner JM, Mahadevaiah SK, Benavente R, Offenberg HH, Heyting C, et al. (2000) Analysis of male meiotic "sex body" proteins during XY female meiosis provides new insights into their functions. Chromosoma 109: 426-432.

11. Toure A, Grigoriev V, Mahadevaiah SK, Rattigan A, Ojarikre OA, et al. (2004) A protein encoded by a member of the multicopy Ssty gene family located on the long arm of the mouse Y chromosome is expressed during sperm development. Genomics 83: 140-147.

12. Akerfelt M, Henriksson E, Laiho A, Vihervaara A, Rautoma K, et al. (2008) Promoter ChIP-chip analysis in mouse testis reveals Y chromosome occupancy by HSF2. Proc Natl Acad Sci U S A 105: 11224-11229.

13. Wu SM, Baxendale V, Chen Y, Pang AL, Stitely T, et al. (2004) Analysis of mouse germ-cell transcriptome at different stages of spermatogenesis by SAGE: biological significance. Genomics 84: 971-981.

14. Garcia MA, Collado M, Munoz-Fontela C, Matheu A, Marcos-Villar L, et al. (2006) Antiviral action of the tumor suppressor ARF. Embo J 25: 4284-4292.

15. Quinn A, Kashimada K, Davidson TL, Ng ET, Chawengsaksophak K, et al. (2014) A site-specific, single-copy transgenesis strategy to identify 5' regulatory sequences of the mouse testis-determining gene Sry. PLoS One 9: e94813.
